# The Association between Serum Uric Acid Levels and the Prevalence of Vulnerable Atherosclerotic Carotid Plaque: A Cross-sectional Study

**DOI:** 10.1038/srep10003

**Published:** 2015-05-11

**Authors:** Qing Li, Yong Zhou, Kehui Dong, Anxin Wang, Xin Yang, Caifeng Zhang, Yi Zhu, Shouling Wu, Xingquan Zhao

**Affiliations:** 1 Department of Neurology, Beijing Tiantan Hospital, Capital Medical University, Beijing 100050, China; 2 Department of Neurology, People’s Hospital of Xinjiang Vygur Autonomous Region, Urumqi 830002, China; 3 Department of General Practice, School of General Practice and Continuing Education, Capital Medical University, Beijing 100069, China; 4 Department of Neurology, Hebei United University Affiliated Hospital, Tangshan 063000, China; 5 Department of Cardiology, Kailuan Hospital, Hebei United University, Tangshan 063000, China

## Abstract

Little is known about the associations between serum uric acid (SUA) levels and atherosclerotic carotid plaque vulnerability. The aim of this study was to assess the associations of SUA levels with the prevalence of vulnerable atherosclerotic carotid plaque in a community-based cohort. In the Asymptomatic Polyvascular Abnormalities Community (APAC) study, cross-sectional data from 2860 Chinese residents who underwent SUA measurement and ultrasonographic assessment of carotid plaque were analyzed. Multivariable logistic regression models were used to assess the associations of SUA levels with presence of vulnerable carotid plaque. After adjustment for potential confounders, SUA levels were significantly associated with the prevalence of vulnerable plaque amongst the middle-aged adults (odds ratio [OR] = 1.19, 95% confidence interval [CI]: 1.11–1.28). Compared to the lowest quartile, quartiles 2, 3 and 4 had a prevalence OR of 1.33 (1.02–1.74), 1.70 (1.27–2.27) and 2.05 (1.53–2.75), respectively, for the presence of vulnerable carotid plaque (p for trend across quartiles < 0.001). In the APAC study, elevated SUA levels were independently associated with the prevalence of vulnerable carotid plaque in middle-aged adults.

Stroke is a significant public health problem worldwide. Particularly in China, stroke has become the leading cause of death and adult disability^1^. One major cause of stroke is the rupture of a vulnerable atherosclerotic plaque leading to thrombosis^2^,^3^. Uno *et al.* concluded that a focal imbalance between prooxidant and antioxidant defense systems induced plaque instability^4^.

Uric acid is the end product of purine metabolism in humans, and it had been shown to mediate inflammation^5^, induce endothelial dysfunction^6^ and stimulate smooth muscle cell proliferation^7^. Patetsios *et al.*^8^ reported a higher concentration of uric acid in atherosclerotic plaque specimens than non-atherosclerotic control specimens, suggesting that serum uric acid (SUA) might play a role in the etiology of atherosclerosis.

High-resolution ultrasound has been a useful tool to quantify atherosclerotic lesions and its vulnerability^9^. Ultrasound assessment is able to determine a vulnerable plaque and its particular features, namely the presence of ulcers, a hypogenic lesion, a thin fibrous cap and a necrotic core located near the surface^10^.

To date, research on the associations between SUA levels and plaque vulnerability are limited^4^,^11^. In addition, most studies are conducted among hospitalized patients. To our knowledge, there is no published study that is focused on community-based population. Therefore, this study examined the associations between SUA levels and plaque vulnerability among a sample of Chinese population from the Asymptomatic Polyvascular Abnormalities Community (APAC) study.

## Results

### Baseline characteristics

The characteristics of study participants are shown in Table 1. Of the 2860 subjects (2048 male and 812 female), the median age was 57.7 years (range: 40–94 years). The median SUA level was 4.98 mg/dL. SUA quartiles were 1.4 to 4.0 mg/dL, 4.1 to 5.0 mg/dL, 5.1 to 6.0 mg/dL and 6.1 to 14.8 mg/dL. Vulnerable carotid plaque was detected in 1483 (51.9%) subjects, with the prevalence of vulnerable plaque in SUA quartile 1 through 4 as 39.7%, 51.7%, 58.3% and 59.1%, respectively. The significant differences among SUA quartiles were in age, sex, prevalence of hypertension, moderate-to-heavy alcohol use, diuretics use, body mass index (BMI), and levels of triglyceride (TG), high-density lipoprotein cholesterol (HDL-C), low-density lipoprotein cholesterol (LDL-C), creatinine, estimated glomerular filtration rate (eGFR).

### Associations between the SUA levels and vulnerable plaque

Table 2 shows the relationship between SUA quartiles and vulnerable plaque estimated with logistic regression models. When SUA was examined as a continuous variable, a positive association between the SUA level and having vulnerable plaque was observed (crude odds ratio [OR] = 1.23, 95% confidence interval [CI]: 1.17–1.30). In all three multivariable-adjusted models, the positive associations between SUA levels and prevalence of vulnerable plaque remained significant. No significant interactions were observed between SUA and sex, hypertension, diabetes, BMI or eGFR in relation to the vulnerable plaque in adjusted models (p > 0.05 for all the interaction terms, data not shown). Significant interactions with age (p = 0.017) were found and analyses were thus stratified by age groups: middle-aged (40 to 59 years) and older (60 to 90 years) adults. In age-stratified analyses, SUA levels were significantly associated with the prevalence of vulnerable plaque amongst the middle-aged adults (adjusted OR = 1.19, 95% CI: 1.11–1.28). In contrast, there was no association in the older age group (adjusted OR = 0.97, 95% CI: 0.88–1.06), suggesting that higher SUA level were significantly associated with an increased risk of vulnerable plaque only among the middle-aged adults but not older adults. Furthermore, a clear dose–response relationship was observed between SUA levels and the presence of vulnerable carotid plaque. The prevalence OR (95% CI) of having vulnerable carotid plaques for middle-aged adults in SUA quartiles 2, 3 and 4 were 1.33 (1.02–1.74), 1.70 (1.27–2.27), and 2.05 (1.53–2.75), respectively, compared to those in quartile 1(p for trend < 0.001).

## Discussion

In this large community-based population, we found elevated SUA levels were associated with a higher prevalence of atherosclerotic vulnerable carotid plaque independently of other atherosclerotic risk factors in middle-aged adults (40 to 59 years old).

Our findings are consistent with previous studies which were conducted in persons with various clinical conditions. Tan *et al.*^11^ reported among 116 stroke-free participants, high SUA levels were associated with an increased risk of vulnerable plaque (OR = 8.36, 95% CI: 1.46–47.90). Hirata *et al.*^12^ found diabetic patients with vulnerable plaque had higher SUA levels compared to patients with stable plaque (p = 0.045). These results indicate that higher SUA levels may be associated with carotid plaque vulnerability in selected populations.

However, not all published literature reported a positive association between SUA levels and carotid plaque vulnerability. Uno *et al.*^4^ measured the uric acid levels of plasma samples and carotid plaque specimens of 35 patients undergoing carotid endarterectomy (CEA). They found no statistical difference in the level of uric acid between stable plaque and vulnerable plaque or in the level of SUA between patients with stable plaque and those with vulnerable plaque. The discrepancy between their findings and ours may be in part due to the differences in participant’s characteristics and assessment tools. Participants in Uno’s study were the patients undergoing CEA and the degree of carotid artery stenosis in these participants might therefore be more serious than ones in our study. Moreover, Uno *et al.*^4^ assessed the carotid plaque vulnerability based on the level of macrophage infiltration and on histopathological findings, which was different from the approach used in our study.

Our analyses revealed a dose–response relationship between SUA levels and prevalence of vulnerable carotid plaque. Hyperuricemia is variably defined as a SUA level greater than either 6.8 or 7.0 mg/dL^13^,^14^. Our results showed that SUA level, even within a normal range (i.e., 4.1–5.0 mg/dL of quartile 2), was associated with an increased prevalence of vulnerable plaque after adjustment for confounding factors. Currently, asymptomatic hyperuricemia is not an indication for initiating urate-lowering therapy^15^. Our finding suggests that it may be necessary to revisit the current guideline for managing asymptomatic hyperuricemia.

There are several potential patho-physiological mechanisms linking SUA to plaque vulnerability. First, inflammation plays a central role in the development of atherosclerosis and plaque vulnerability^16^,^17^. Uric acid can induce inflammatory pathways in rat vascular smooth muscle cells in vitro^5^, and inflammation can elicit acute plaque rupture that results in stroke^18^. Second, SUA might contribute to the activation of local platelets and formation of mural thrombosis^19^, which are associated with an elevated risk of vulnerable plaque. Third, intimal calcification of atherosclerotic plaques is considered a risk factor for plaque rupture^20^,^21^. Animal experiments revealed that strong positive correlations exist between the size of granules of calcium salts and uric acid level (*r* ranges from 0.64 to 0.66)^22^. These mechanisms may potentially contribute to the development of vulnerably plaque observed in subjects with higher SUA levels. We found that the associations between SUA and vulnerable carotid plaque were significant in middle-aged adults but not in older adults. We are unclear about the underlying mechanisms that may explain this age difference.

Our study has several limitations. First, this is a cross-sectional observational study rather than an interventional study, so a causal inference may not be drawn. Second, our participants were middle-aged or older adults, therefore the findings may not apply to younger subjects. Third, a history of gout and the use of medications to treat hyperuricemia or gout were not recorded in our study. If the diseases and/or their treatments impact the formation of vulnerable carotid plaque, our results are biased by residual confounding. Fourth, the study participants were individuals from China, so our results may not be generalizable to other populations. Lastly, the assessment of vulnerable plaque with ultrasound may be less reliable than the high-resolution magnetic resonance imaging.

In summary, we found positive associations between SUA levels and the prevalence of vulnerable atherosclerotic carotid plaque among mid-aged Chinese residents after adjustment for age, sex, smoking status, eGFR, diuretics use and statins use. The elevated risk of vulnerable plaque formation was found among subjects with a SUA level between 4.1 and 14.8 mg/dL. This result suggests that elevated SUA may have a pathophysiologic role in the formation of vulnerable carotid plaque in Chinese middle-aged adults.

## Methods

### Study design and population

The APAC study is a community-based observational study which is aimed to investigate the epidemiology of asymptomatic intracranial atherosclerotic stenosis, carotid atherosclerosis and peripheral artery diseases in Chinese adults. The methods of subject recruitment and enrollment have been described in details elsewhere^23^. Briefly, a total of 5440 subjects in 2010 were enrolled from the participants of the Kailuan study^24^. Inclusion criteria were (1) aged ≥40 years, and (2) free of stroke, transient ischemic attack (TIA), or coronary disease. All participants signed their informed consent forms. Based on a cross-sectional design, this study included a sample of APAC participants who had valid SUA measures and an ultrasound evaluation of carotid plaque vulnerability. Among 5440 APAC subjects, carotid plaque was detected in 2944 participants, of which 2860 had SUA measurement.

### Measurement of indicators

Well-trained interviewers administered a standardized and structured questionnaire to gather information on subjects’ demographic characteristics, vascular risk factors and medical conditions. Physical examination was performed by physicians. Smoking status was classified as “non-smoking” or “smoking”, based on self-reported information. A standard drink defined as one that contains roughly 14 grams of pure alcohol (355 milliliters of regular beer, 147.9 milliliters of wine, or 44.4 milliliters of distilled spirits). Taking ≥2 drinks per day was defined as moderate-to-heavy alcohol use. Methods for measurement of blood pressure, BMI, collection of fasting blood specimens for measuring glucose and lipids, and the definitions of other vascular risk factor covariates in APAC study have been described previously^23^. Dyslipidemia was defined as LDL-C ≥ 130 mg/dL, HDL-C < 40 mg/dL, TG ≥ 150 mg/dL, or current treatment with lipid-lowering therapy^25^. Serum creatinine was measured with Jaffe’s kinetic method. eGFR was calculated with an equation adapted from the Modification of Diet in Renal Disease (MDRD) equation^26^.

### Assessment of SUA

Venous blood samples were obtained via venipuncture of the large antecubital veins after overnight fasting. SUA levels were determined with Uricase-Peroxidase method using an autoanalyzer (Hitachi 747; Hitachi, Tokyo, Japan) at the central laboratory of the Kailuan General Hospital.

### Assessment of vulnerable carotid plaque

Plaque was assessed by sonographers blinded to participants’ clinical characteristics including the SUA level. High-resolution B-mode ultrasounds (Philips iU-22 ultrasound system, Philips Medical Systems, Bothell, WA) with a 5–12 MHz linear array transducer was used to detect plaques bilaterally on 3 segments: the common carotid artery, the bifurcation and the internal carotid artery. Intima-media thickness (IMT) was defined as the distance from the leading edge of the lumen-intima interface to the leading edge of the media-adventia interface on a longitudinal image of each carotid artery. Atherosclerotic plaque was defined as a focal structure encroaching into the arterial lumen of at least 0.5 mm or 50% of the surrounding IMT value, or a thickness >1.5 mm as measured from the media-adventitia interface to the intima-lumen interface^27^. The plaque vulnerability is determined based on morphological ultrasound characteristics. In this study, as previously described^28^, vulnerable plaque was defined as the presence of surface irregularity or ulceration, or anechogenic plaque, or heterogeneous plaque^10^.

### Statistical analysis

Statistical analyses were performed using SPSS for Windows 21.0 (SPSS Inc., Chicago IL, USA). Continuous data were presented as median and inter-quartile range (IQR) because of skewed distributions, and categorical variables were presented as counts and percentages. We classified the participants into 4 groups according to SUA quartiles. As data were non-normally distributed, group differences in clinical and demographic characteristics were analyzed using chi-square test or Kruskal-Wallis tests.

Multivariable logistic regression models were constructed to examine the associations between SUA levels (continuous and in quartiles) with the prevalence of vulnerable plaque after adjusting for potential confounders. We started with a parsimonious model (i.e., model 1) that only adjusted for age, sex, smoking status and eGFR, which were found significant predictors of vulnerable plaque. In model 2, we further adjusted for use of diuretics and use of statins because their potential impacts on SUA level and plaque vulnerability. To make sure that our results were not biased by residual confounding from measured covariates, we also constructed model 3 that adjusted for all measured potential confounders, including age, sex, moderate-to-heavy alcohol use, hypertension, diuretics use, BMI, dyslipidemia and eGFR.

Trend test was performed to examine whether there was a dose-response relationship between SUA quartiles and plaque vulnerability. Potential effect-measure modification was also assessed with age, sex, hypertension, diabetes, BMI, dyslipidemia and eGFR. If the interaction term was statistically significant, the stratified analysis would be performed. A probability value <0.05 (2-tailed) was considered significant.

### Ethical approval

Ethical approvals for the APAC study protocol were obtained from the ethics committees of the Kailuan General Hospital and Beijing Tiantan Hospital. All the experiments described here were performed in accordance with the approved guidelines.

## Author Contributions

L.Q., Yo.Z., S.L.W. and X.Q.Z. conceived and designed the study; K.H.D., A.X.W., X.Y., C.F.Z. and Yi.Z analyzed and interpreted the data; L.Q., Yo.Z. and K.H.D. drafted the manuscript. All authors revised the manuscripts for important intellectual content.

## Additional Information

**How to cite this article**: Li, Q. *et al.* The Association between Serum Uric Acid Levels and the Prevalence of Vulnerable Atherosclerotic Carotid Plaque: A Cross-sectional Study. *Sci. Rep.* **5**, 10003; doi: 10.1038/srep10003 (2015).

## Figures and Tables

**Table 1:** Demographic and clinical characteristics by SUA quartiles.

	**Total**	**SUA**				**p-value**
		**Quartiles 1**	**Quartiles 2**	**Quartiles 3**	**Quartiles 4**	
SUA range (median), mg/dL	1.4–14.8 (4.98)	1.4–4.0 (3.38)	4.1–5.0 (4.61)	5.1–6.0 (5.48)	6.1–14.8 (6.91)	
Number of subjects	2860	770	714	665	711	
Male, n (%)	2048 (71.6)	418 (54.3)	493 (69.0)	525 (78.9)	612 (86.1)	<0.001
Age, y	57.7 (50.0–70.1)	52.6 (46.7-59.6)	57.0 (50.0–68.6)	62.1 (53.0–72.1)	62.2 (52.4–75.0)	<0.001
Smoking status, n (%)	1064 (37.2)	258 (33.5)	279 (39.1)	261 (39.2)	266 (37.4)	0.079
Moderate -to- heavy alcohol use, n (%)	432 (15.1)	96 (12.5)	99 (13.9)	106 (15.9)	131 (18.4)	0.009
Hypertension, n (%)	1696 (59.3)	404 (52.5)	404 (56.6)	404 (60.8)	484 (68.1)	<0.001
Diabetes mellitus, n (%)	467 (16.3)	143 (18.6)	102 (14.3)	112 (16.8)	110 (15.5)	0.137
Dyslipidemia, n (%)	1288 (45.0)	272 (35.3)	314 (44.0)	305 (45.9)	397 (55.8)	<0.001
Diuretics use, n (%)	77 (2.7)	16 (2.1)	13 (1.8)	12 (1.8)	36 (5.1)	<0.001
Statins use, n (%)	22 (0.8)	6 (0.8)	5 (0.7)	3 (0.5)	8 (1.1)	0.55
BMI, kg/m^2^	24.8 (22.7–27.1)	24.2 (22.2–26.5)	25.5(22.5–26.7)	24.8(22.8–27.0)	25.6(23.5–27.7)	<0.001
TG, mg/dL	119.5 (85.9–173.3)	111.6 (84.1–151.4)	113.3 (82.4–163.8)	119.5 (83.7–172.2)	136.4 (97.4–215.2)	<0.001
HDL-C, mg/dL	59.9 (49.9–72.7)	62.6 (51.4–76.2)	60.7 (49.1–74.2)	58.8 (49.5–71.9)	56.8 (48.3–67,7)	<0.001
LDL-C, mg/dL	103.1 (85.5–120.7)	104.4 (90.0–120.7)	103.8 (85.1–121.0)	101.7 (84.1–120.7)	100.5 (81.6–119.9)	0.027
Creatinine, mg/dL	0.83 (0.72–0.97)	0.79 (0.69–0.91)	0.81 (0.70–0.94)	0.86 (0.74–1.0)	0.88 (0.78–1.02)	<0.001
eGFR (mL/min/1.73m^2^)	100.4 (82.9–117.1)	105.3 (87.3–121.2)	102.7 (85.2–118.9)	98.7 (82.7–116.3)	94.7 (78.6–110.1)	<0.001
Prevalence of vulnerable plaque, n (%)	1483 (51.9)	306 (39.7)	369 (51.7)	388 (58.3)	420 (59.1)	<0.001

Values are expressed as the median and inter-quartile range (IQR), or numbers (percentage).

SUA indicates serum uric acid; BMI, body mass index; TG, triglyceridese; HDL-C, high-density lipoprotein cholesterol; LDL-C, low-density lipoprotein cholesterol; eGFR, estimated glomerular filtration rate.

**Table 2:** OR and 95% CI between SUA levels and prevalence of vulnerable atherosclerotic carotid plaque.

	**SUA**						
	**Quartiles 1**	**Quartiles 2**	**Quartiles 3**	**Quartiles 4**	**Continuous Scale**	**p for trend**	**p interaction**
	**1.4–4.0 mg/dL**	**4.1–5.0 mg/dL**	**5.1–6.0 mg/dL**	**6.1–14.8 mg/dL**			
Crude Model	Ref	1.62 (1.32–1.99)	2.12 (1.72–2.62)	2.19 (1.78–2.69)	1.23 (1.17–1.30)	<0.001	
Model 1^1^	Ref	1.27 (1.02–1.58)	1.42 (1.13–1.78)	1.41 (1.13–1.78)	1.11 (1.05–1.17)	0.002	
Model 2^2^	Ref	1.27 (1.02–1.58)	1.43 (1.14–1.79)	1.42 (1.13–1.78)	1.10 (1.05–1.17)	0.002	
Model 3^3^	Ref	1.27 (1.02–1.58)	1.41 (1.12–1.77)	1.37 (1.09–1.74)	1.10 (1.04–1.16)	0.006	
Age-stratified analyses							0.017
40–59 (year)^4^	Ref	1.33 (1.02–1.74)	1.70 (1.27–2.27)	2.05 (1.53–2.75)	1.19 (1.11–1.28)	<0.001	
≧ 60 (year)^5^	Ref	1.02 (0.69–1.52)	0.95 (0.64–1.41)	0.77 (0.52–1.14)	0.97 (0.88–1.06)	0.112	

Data expressed by odds ratio (95% confidence interval) and p value.

OR indicates odds ratio; CI, confidence interval.

^1^Adjusted for age, sex, smoking status and eGFR.

^2^Adjusted for age, sex, smoking status, eGFR, diuretics use and statins use.

^3^Adjusted for age, sex, moderate-to-heavy alcohol use, hypertension, diuretics use, BMI, dyslipidemia and eGFR.

^4^Adjusted for sex, smoking status, eGFR, diuretics use and statins use.

^5^Adjusted for sex, smoking status, eGFR, diuretics use and statins use.
